# Prognostic impact of in vivo soluble cell adhesion molecules in metastatic renal cell carcinoma

**DOI:** 10.1038/sj.bjc.6690277

**Published:** 1999-04

**Authors:** R Hoffmann, A Franzke, J Buer, S Sel, K Oevermann, A Duensing, M Probst, S Duensing, H Kirchner, A Ganser, J Atzpodien

**Affiliations:** Department of Hematology and Oncology and Department of Pathology, Medizinische Hochschule Hannover, Carl-Neuberg-Str. 1, Hannover, 30625, Germany; Department of Cell Biology and Immunbiology, National Centre for Biotechnology (OBF), Mascheroder Weg 1, Braunschweig, Germany

**Keywords:** renal carcinoma, soluble adhesion molecules, predictor

## Abstract

The purpose of the study was to determine prognostic significance of pretreatment serum levels of different molecules involved in cell to cell interactions along with other clinical parameters in patients with metastatic renal cell carcinoma. sICAM-1, sVCAM-1 and sELAM-1 serum levels were determined by ELISA assays in sera from 99 patients with histologically confirmed progressive metastatic renal cell carcinoma prior to initiation of systemic therapy. Kaplan–Meier survival analysis, log-rank statistics and two-proportional Cox regression analyses were employed to identify risk factors and to demonstrate statistical independence. In univariate analyses, the following pretreatment risk factors could be identified: serum sICAM-1 level > 360 ng ml^−1^, erythrocyte sedimentation rate > 70 mm h^−1^, serum C-reactive protein level > 8 mg l^−1^, serum lactic dehydrogenase level > 240 U/ l and neutrophil count > 6000 μ^l−;1^. Multivariate analyses demonstrated statistical independence for serum sICAM-1 level, erythrocyte sedimentation rate (ESR) and serum C-reactive protein (CRP) level as pretreatment predictors of overall patient survival. The prognostic significance of sICAM-1 might indicate a role of this molecule for tumour progression, potentially in association with the abrogation of anti-tumour immune responses. The possibility of defining a pretreatment risk model based on sICAM-1 level, ESR and CRP also warrants further investigation, with regard to a possible linkage between acute phase proteins and sICAM-1 levels. © 1999 Cancer Research Campaign


*British Journal of Cancer* (1999) **79**(11/12), 1742–1745
© 1999 Cancer Research Campaign
Article no. bjoc.1998.0277

### Prognostic impact of in vivo soluble cell adhesion molecules in metastatic renal cell carcinoma

**R Hoffmann[1], A Franzke[1], J Buer[2], S Sel[1], K Oevermann[1], A Duensing[1], M Probst[1], S Duensing[1], H Kirchner[1], A Ganser[1] and J Atzpodien[1]**

[1]Medizinische Hochschule Hannover, Department of Hematology and Oncology and Department of Pathology, Carl-Neuberg-Str. 1, 30625 Hannover, Germany; [2]Department of Cell Biology and Immunbiology, National Centre for Biotechnology (OBF), Mascheroder Weg 1, Braunschweig, Germany

**Summary** The purpose of the study was to determine prognostic significance of pretreatment serum levels of different molecules involved in cell to cell interactions along with other clinical parameters in patients with metastatic renal cell carcinoma. sICAM-1, sVCAM-1 and sELAM-1 serum levels were determined by ELISA assays in sera from 99 patients with histologically confirmed progressive metastatic renal cell carcinoma prior to initiation of systemic therapy. Kaplan–Meier survival analysis, log-rank statistics and two-proportional Cox regression analyses were employed to identify risk factors and to demonstrate statistical independence. In univariate analyses, the following pretreatment risk factors could be identified: serum sICAM-1 level > 360 ng $ml^{-1}$, erythrocyte sedimentation rate > 70 mm $h^{-1}$, serum C-reactive protein level > 8 mg $l^{-1}$, serum lactic dehydrogenase level > 240 U/ l and neutrophil count > 6000 $\mu^{l-1}$. Multivariate analyses demonstrated statistical independence for serum sICAM-1 level, erythrocyte sedimentation rate (ESR) and serum C-reactive protein (CRP) level as pretreatment predictors of overall patient survival. The prognostic significance of sICAM-1 might indicate a role of this molecule for tumour progression, potentially in association with the abrogation of anti-tumour immune responses. The possibility of defining a pretreatment risk model based on sICAM-1 level, ESR and CRP also warrants further investigation, with regard to a possible linkage between acute phase proteins and sICAM-1 levels.

**Keywords:** renal carcinoma; soluble adhesion molecules; predictor

Renal cell carcinoma is an indolent disease diagnosed with distant metastases in a significant proportion of patients. It is usually resistant to chemotherapy; a significant improvement in treatment could be achieved by introduction of cytokines into treatment regimens (Atzpodien et al, 1990). The molecular mechanisms of metastases formation are largely unknown.

Cell adhesion molecules are cell surface receptors involved in leucocyte homing, adhesion and migration. Although these proteins are usually membrane-bound, soluble forms exist and are generated by shedding of the extracellular portions from the cell surface (Rothlein et al, 1991).

Intercellular adhesion molecule-1 (ICAM-1) and vascular cell adhesion molecule 1 (VCAM-1) belong to the immunoglobulin superfamily and are expressed on a wide variety of cells including endothelia, antigen-presenting cells and B-cells (Roit et al, 1996; van den Stolpe and van der Saag, 1996). The ICAM-1-ligand, LFA-1 (Marlinn and Springer, 1987; Roit et al, 1996), is expressed on most leukocytes, including T-cells and NK-cells; the VCAM-1 ligand, VLA-4, is primarily expressed on lymphocytes. These ligands belong to the integrin superfamily (Roit et al, 1996). E-selectin (ELAM-1) is expressed on endothelium and its N-terminal lectin domain binds to carbohydrate groups associated with a variety of proteins of leukocyte cell surfaces, like the CD15 associated Lewis-X carbohydrate group (Roit et al, 1996).

Prior studies have demonstrated significant differences in expression of VLA-integrins between normal kidney tissue and low/high grade renal cell carcinoma specimens (Anastassiou et al, 1995). Another example for a possible relevance of cell adhesion molecule expression in the course of malignant disease is the fact that endothelial and leucocyte expression of PECAM-1 has shown impact on leucocyte infiltration of the tumour tissue and on patient survival (Anastassiou et al, 1995). However, it remains unclear whether serum levels of the soluble isoforms are of prognostic value. To test this hypothesis in advanced renal cell carcinoma, we examined sera from 99 patients for pretreatment serum levels of sICAM-1, sVCAM-1 and sELAM-1.

*Received 19 May 1998*
*Revised 28 July 1998*
*Accepted 19 August 1998*

*Correspondence to:* J Atzpodien

### PATIENTS AND METHODS

### Patient selection and treatment

Between 1989 and 1996, 99 patients were assigned to receive either immunotherapy or combined chemo-/immunotherapy according to the following schedule:

Interferon-α subcutaneously, 5 MIU $m^{-2}$ on day 1 of weeks 1 and 4, on days 1, 3, 5 of weeks 2 and 3; 10 MIU $m^{-2}$ on days 1, 3, 5 of weeks 5–8. Interleukin-2 subcutaneously, 20 MIU $m^2$, on days 3, 4, 5 of weeks 1 and 4, and 5 MIU $m^{-2}$ on days 1, 3, 5 of weeks 2 and 3. Additionally, for patients in the combined chemo-/immunotherapy group, 5-fluorouracil was administered on day 1 of weeks 5–8 as a 1000 mg $m^{-2}$ intravenous infusion. Concomitant medication was given as needed to control adverse effects of immunotherapy.

All patients had histologically (by nephrectomy) confirmed metastatic renal cell carcinoma in progressive state and a Karnofsky performance status of ≥ 70%. Written informed consent was obtained from each patient prior to administration of any

**Table 1** Patient characteristics and risk categories according to elevated serum levels of soluble ICAM-1

| Characteristic | All patients | sICAM-1 < 360 ng ml | sICAM-1 ≥ 360 ng ml |
|---|---|---|---|
| Entered | 99 | 87 | 11 |
| Sex | | | |
| Male | 73 | 66 | 7 |
| Female | 26 | 22 | 4 |
| Age (years) | | | |
| Median | 58 | 58 | 52 |
| Range | 32–74 | 32–74 | 40–68 |
| Metastases | | | |
| Bone | 29 | 22 | 7 |
| Brain | 15 | 14 | 1 |
| Liver | 21 | 19 | 2 |
| Lung | 74 | 65 | 9 |
| Other | 52 | 45 | 7 |
| Treatment response | | | |
| Complete response | 11 | 11 | 0 |
| Partial remission | 28 | 27 | 1 |
| Stable disease | 45 | 38 | 7 |
| Progressive disease | 15 | 12 | 3 |
| Median survival (months) | 23 | 26 | 8 |
| 5-Year survival (%) | 22 | 26 | 0 |

study medication; response and survival were assessed according to World Health Organization (WHO) criteria. Table 1 shows the patient characteristics.

### Sample collection and soluble cell adhesion molecule serum level measurements

Serum samples were obtained from each patient prior to initiation of therapy and stored at – 70°C until analysis. Soluble cell adhesion molecule serum levels were determined by enzyme-linked immunosorbent assay (ELISA) as follows: sVCAM-1, Medgenix, Ratingen, Germany; sICAM-1 and sELAM-1, Quantikine, R&D, Wiesbaden, Germany. All analyses were performed in duplicate, and sera were diluted to reach a concentration within the standard curve of the respective assay, when necessary.

As shown previously (Franzke et al, 1998), analysis of sera from 10 healthy control subjects showed mean serum values ± SEM for sVCAM-1, sICAM-1 and sELAM-1 of 509 ng $ml^{-1}$ ± 14 ng $ml^{-1}$, 208 ng $ml^{-1}$ ± 12 ng $ml^{-1}$ and 54 ng $ml^{-1}$ ± 7 ng $ml^{-1}$, respectively.

### Statistical analysis

The statistical end point was overall survival from treatment start. Survival analysis and univariate risk factor analysis were performed according to the method of Kaplan and Meier, and multvariate risk factor analysis according to the two-proportional Cox regression analysis with forward selection of variables. Impact of potential risk factors on survival was calculated with log-rank statistics. Clinical parameters and soluble cell adhesion molecules were tested as dichotomized variables, defining the cut-off value as the value that best discriminates between good and poor overall survival.

### RESULTS

### Overall survival and univariate risk factor analysis

Median survival of all patients entered into the study was 23 months, with 35 patients being alive at last follow-up. Survival ranged from 1 to 80+ months. As shown in Table 2, of all cell adhesion molecule serum levels assayed, only sICAM-1 showed a predictive significance, with a median survival of 27 months in patients with pretreatment sICAM-1 serum levels below 360 ng $ml^{-1}$ as opposed to a median survival of 8 months in patients with elevated pretreatment sICAM-1 serum levels ($P = 0.0011$).

In addition, the following pretreatment clinical factors could be identified as univariate predictors of overall survival: elevated erythrocyte sedimentation rate (ESR) ($P = 0.001$; median survival, 30 vs 10 months), elevated serum C-reactive protein (CRP) ($P = 0.0001$; median survival, 37 vs 15 months), elevated serum lactic dehydrogenase ($P = 0.0158$; median survival, 25 vs 8 months) and

**Table 2** Pretreatment clinical factors and their prognostic significance as determined by univariate Kaplan–Meier analysis

| Risk factor | Categories compared | *P*-value |
|---|---|---|
| Clinical features | | |
| Age | < 60 vs ≥ 60 years | 0.1545 |
| Sex | Male vs Female | 0.6317 |
| ESR | < 70 vs ≥ 70 mm $h^{-1}$ | 0.001 |
| CRP | < 8 vs ≥ 8 mg $ml^{-1}$ | 0.0001 |
| Haemoglobin level | < 10 vs ≥ 10 g $dl^{-1}$ | 0.0773 |
| Absolute neutrophil count | ≤ 6000 vs > 6000 $\mu l^{-1}$ | 0.0025 |
| Serum lactic dehydrogenase | < 240 vs ≥ 240 U $l^{-1}$ | 0.0158 |
| Time from diagnosis to first occurrence of metastases | ≤ 12 vs > 12 months | 0.1047 |
| Time from first occurrence of metastases to treatment start | < 10 vs ≥ 10 months | 0.3697 |
| Osseous metastases | Absent vs present | 0.4187 |
| Cerebral metastases | Absent vs present | 0.0603 |
| Hepatic metastases | Absent vs present | 0.8593 |
| Pulmonary metastases | Absent vs present | 0.9783 |
| Adhesion molecules | | |
| sICAM-1 | < 360 vs ≥ 360 ng $ml^{-1}$ | 0.0011 |
| sELAM-1 | < 60 vs ≥ 60 ng $ml^{-1}$ | 0.1004 |
| sVCAM-1 | < 770 vs ≥ 770 ng $ml^{-1}$ | 0.7970 |

© Cancer Research Campaign 1999

**Table 3** Statistically independent risk factors identified by multivariate Cox regression analysis

| Risk factor | Categories compared | *P*-value |
|---|---|---|
| Serum sICAM-1 level | < 360 ng ml$^{-1}$ vs ≥ 360 ng ml$^{-1}$ | 0.0092 |
| Serum C-reactive protein | < 8 mg l$^{-1}$ vs ≥ 8 mg l$^{-1}$ | 0.0164 |
| Erythrocyte sedimentation rate | < 70 mm 1 h$^{-1}$ vs ≥ 70 mm 1 h$^{-1}$ | 0.0226 |

elevated absolute neutrophil count ($P$ = 0.0025; median survival, 28 vs 11 months). No predictive power could be shown for sVCAM-1 serum level, sELAM-1 serum level, sex, age, haemoglobin level, time from diagnosis to first occurrence of metastases, or time from first occurrence of metastases to treatment start. Also, the presence or absence of osseous, cerebral, hepatic, or pulmonary metastases had no prognostic value.

### Multivariate risk factor analysis

Among all pretreatment risk factors identified by univariate analysis, a multivariate Cox regression analysis was performed to identify statistically independent predictors of survival. Elevated serum sICAM-1 level, ESR and serum CRP were found to be significant and statistically independent (Table 3). Of these predictors, sICAM-1 level showed a particular power to discriminate between good risk and poor risk patients ($P$ = 0.0092).

Comparing 5-year survival rates, patients with low (< 360 ng ml$^{-1}$) sICAM-1 levels and patients with elevated (≥ 360 ng ml$^{-1}$) sICAM-1 levels reached 26% and 0%, respectively. Also, response rates differed significantly between these two groups: all patients achieving complete responses had low pretreatment sICAM-1 levels (Table 1).

### DISCUSSION

The present study examined prognostic significance of pretreatment soluble cell adhesion molecule serum levels and other clinical laboratory parameters in unselected metastatic renal cell carcinoma patients. Serum sICAM-1 levels, ESR and serum CRP levels could be identified as highly significant and statistically independent predictors of overall survival. While ESR and CRP are rather unspecific indicators of inflammatory activity, sICAM-1 expression needs closer attention.

ICAM-1 has been reported to be aberrantly expressed on cells from a variety of human malignancies: myeloid leukaemias (Maio et al, 1990), B-cell lymphoproliferative disorders (Maio et al, 1990), neuroblastoma (Foreman et al, 1993), non-small-cell lung cancer (NSCLC) (Passlick et al, 1994), prostate carcinoma (Wolff et al, 1995) and head and neck squamous cell carcinoma (Vora et al, 1997). While its expression on tumour cells is generally linked to poor prognosis, in NSCLC it appears to be rather expressed by well-differentiated and localized tumours (Passlick et al, 1994). Particular attention has been directed to ICAM-1 expression in malignant melanoma, where it can be demonstrated in the majority of cases. Increased expression in metastases as compared to the primary tumour has been demonstrated, suggesting a role in the formation of metastases (Natali et al, 1990). Furthermore, ICAM-1-positive primary melanomas tend to recur after surgical excision (Natali et al, 1997). Surprisingly, these features seemed to be unique to melanoma and could not be reproduced in a variety of

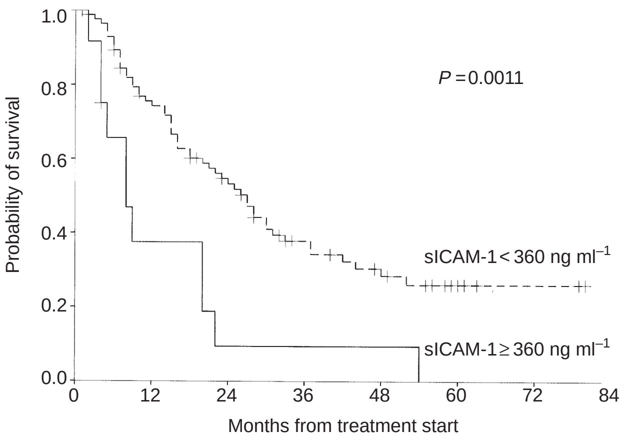

**Figure 1** Pretreatment sICAM-1 serum levels and survival in 99 patients with metastatic renal cell carcinoma. Survival curves (Kaplan–Meier estimate) of patients with either low (< 360 ng ml$^{-1}$) or elevated (≥ 360 ng ml) pretreatment serum levels of sICAM-1. *P*-value was determined by log-rank test. Tick marks represent patients for whom data were censored ($n$ = 34 and $n$ = 1, for patients with low or elevated pretreatment serum sICAM-1 levels, respectively)

malignancies from different embryonal origins (Natali et al, 1990) and in renal cell carcinoma (Heicapell et al, 1994).

Not only the expression of ICAM-1 on tumour cells has demonstrated prognostic significance, but also the level of the soluble form as measured in patients' sera. This has been demonstrated for acute lymphoblastic leukaemia (Hatzistilianou et al, 1997), malignant melanoma (Schadendorf et al, 1996), non-Hodgkins lymphoma (Christiansen et al, 1996), Hodgkin's disease (Christiansen et al, 1995) and hepatocellular carcinoma (Shimizu et al, 1995), amongst others.

We demonstrated the prognostic significance and independence not only for pretreatment sICAM-1 level, but also for ESR and CRP. Notably, elevated serum sICAM-1 by itself was capable of identifying patients at highest risk, i.e. with a 5-year survival probability of 0%. Although we could demonstrate statistical independence of sICAM-1 levels and each of the above acute phase reactants, earlier studies demonstrated a possible correlation between acute phase reactants and sICAM-1 levels (Reinhardt et al, 1996) or between ESR and serum CRP level (Ljungberg et al, 1990).

Since earlier studies have shown soluble ICAM-1 to be active in influencing the cell-surface ICAM-1/LFA-1 interaction (Welder et al, 1993; Meyer et al, 1995), another issue is that of a potential role of sICAM-1 in formation of metastases and tumour progression, leading to impaired prognosis.

It might be hypothesized that ICAM-1 on circulating tumour cells results in attachment to the capillary endothelium via specific ligands, which in this case still need to be defined. This attachment of cells might ultimately lead to formation of metastases. Since the soluble form of ICAM-1 is produced by shedding of the extracellular portion of the membrane-bound form, it seems likely that high sICAM-1 levels can be found in patients whose tumours express large amounts of surface ICAM-1. However, in that scenario, high sICAM-1 serum levels would occupy the endothelial receptors, preventing tumour cells from adherence and metastases formation.

ICAM-1 is not only involved in cell homing and migration, but also in various cell-to-cell interactions with immunological

 © Cancer Research Campaign 1999

significance, such as T-cell-mediated cytotoxicity (Makgoba et al, 1998), NK-cell-mediated cytotoxicity (Chong et al, 1994), B–T-cell interaction (Boyl et al, 1998; Amblard et al, 1994), T-cell adhesion (Sung et al, 1992), or B-cell activation (Moy and Brian, 1992). Hence, another scenario seems possible: by binding to its ligands on immune effector cells, like LFA-1 on T-cells, the soluble form could effectively inhibit different anti-tumour immune effects like effector mechanisms of cytotoxic T-cell or NK-cells or antigen-dependent respectively T-cell-dependent B-cell activation; this could be part of an immune escape mechanism of tumour cells, and could possibly explain the coincidence of high serum sICAM-1 levels and impaired prognosis. Some of these effects have already been demonstrated in vitro (Fecondo et al, 1991; Becker et al, 1993), underlining the possibility of a specific action of ICAM-1 in the immune escape of malignant cells. Although further investigation is needed, this could clearly be a target for future therapeutic trials.

### REFERENCES

Amblard F, Auffray C, Sekaly R and Fischer A (1994) Molecular analysis of antigen-independent adhesion forces between T and B lymphocytes. *Proc Natl Acad Sci USA* **91**: 3628–3632

Anastassiou G, Duensing S, Steinhoff G, Kirchner H, Ganser A and Atzpodien J (1995) In vivo distribution of integrins in renal cell carcinoma: integrin-phenotype alteration in different degrees of tumor differentiation and VLA-2 involvement in tumor metastases. *Cancer Biother* **10**: 287–292

Atzpodien J, Körfer A, Franks C, Poliwoda H and Kirchner H (1990) Home therapy with recombinant interleukin-2 and interferon-alpha 2b in advanced human malignancies. *Lancet* **335**: 1509

Becker JC, Termeer C, Schmidt RE and Bröcker E-B (1993) Soluble intercellular adhesion molecule 1 inhibits MHC-restricted specific T cell/tumor interaction. *J Immunol* **151**: 7224–7232

Boyd AW, Wawryk SO, Burns GF and Fecondo JV (1988) Intercellular adhesion molecule 1 (ICAM-1) has a central role in cell–cell contact mediated immune mechanisms. *Proc Natl Acad Sci USA* **85**: 3095–3099

Chong AS, Boussy IA, Jiang XL, Lamas M and Graf LH Jr (1994) CD54/ICAM-1 is a costimulator of NK-mediated cytotoxicity. *Cell Immunol* **157**: 92–105

Christiansen I, Enblad G, Kalkner KM, Gidlof C, Glimelius B and Töttermann T (1995) Soluble ICAM-1 in Hodgkins disease: a promising independent predictive marker for survival. *Leuk Lymphoma* **19**: 243–251

Christiansen I, Gidlof C, Kalkner KM, Hagberg H, Bennmarker H and Tötermann T (1996) Elevated serum levels of soluble ICAM-1 in non-Hodgkins lymphoma correlates with tumor burdon, clinical activity, and other prognostic markers. *Br J Haematol* **92**: 639–646

Fecondo JV, Kent SB and Boyd AW (1991) Inhibition of intercellular adhesion molecule 1 dependent biological activities by a synthetic peptide analog. *Proc Natl Acad Sci USA* **88**: 2879–2882

Foreman NK, Rill DR, Coustan-Smith E, Douglass EC and Brenner MK (1993) Mechanisms of selective killing of neuroblastoma cells by natural killer cells and lymphokine activated killer cells. Potential for minimal disease eradication. *Br J Cancer* **67**: 933–938

Franzke A, Probst-Kepper M, Buer J, Duensing S, Hoffmann R, Wittke F, Volkenandt M, Ganser A and Atzpodien J (1998) Elevated pretreatment serum levels of soluble vascular cell adhesion molecule 1 and lactate dehydrogenase as predictors of survival in cutaneous metastatic malignant melanoma. *Br J Cancer* **78**: 40–45

Hatzistilianou M, Athanassiadou F, Agguridaki C and Catriu D (1997) Circulating soluble adhesion molecule levels in children with acute lymphoblastic leukemia. *Eur J Pediatr* **156**: 537–540

Heicapell R, Podlinski J, Buszello H and Ackermann R (1994) Cell surface expression and serum levels of intercellular adhesion molecule 1 in renal cell carcinoma. *Urol Res* **22**: 9–15

Ljungberg B, Grankvist K and Rasmuson T (1995) Acute phase reactants and prognosis in renal cell carcinoma. *Cancer* **76**: 1435–1439

Maio M, Pinto A, Carbone A, Zagonel V, Gloghini A, Marotta G, Cirillo D, Colombatti A, Ferrara F and Del-Vecchio L (1990) Differential expression of CD54/intercellular adhesion molecule 1 im myeloid leukemias and lymphoproliferative disorders. *Blood* **76**: 783–790

Makgoba MW, Sanders ME, Ginther Luce GE, Gugel EA, Dustin ML, Springer TA and Shaw S (1988) Functional evidence that intercellular adhesion molecule 1 (ICAM-1) is a ligand for LFA-1 dependent adhesion in T-cell mediated cytotoxicity. *Eur J Immunol* **18**: 637–640

Marlin SD and Springer TA (1987) Purified intercellular adhesion molecule 1 (ICAM-1) is a ligand for lymphocyte function-associated antigen 1 (LFA-1). *Cell* **51**: 813–819

Meyer DM, Dustin ML and Carron CP (1995) Characterization of intercellular adhesion molecule 1 ectodomain (sICAM-1) as an inhibitor of lymphocyte function associated molecule 1 interaction with ICAM-1. *J Immunol* **155**: 3578–3584

Moy VT and Brian AA (1992) Signaling by lymphocyte function associated antigen 1 (LFA-1) in B-cells: enhanced antigen presentation after stimulation through LFA-1. *J Exp Med* **175**: 1–7

Natali P, Nicotra MR, Cavaliere R, Bigotti A, Romano G, Temponi M and Ferrone S (1990) Differential expression of intercellular adhesion molecule 1 in primary and metastatic melanoma lesions. *Cancer Res* **50**: 1271–1278

Natali PG, Hamby CV, Felding-Habermann B, Liang B, Nicotra MR, Di-Filippo F, Giannarelli D, Temponi M and Ferrone S (1997) Clinical significance of alpha(v)beta3 integrin and intercellular adhesion molecule 1 expression in cutaneous malignant melanoma lesions. *Cancer Res* **57**: 1554–1560

Passlick B, Izbicki JR, Simmel S, Kubuschok B, Karg O, Habekost M, Thetter O, Schweiberer L and Pantel K (1994) Expression of major histocompatibility class I and class II antigens and intercellular adhesion molecule 1 on operable non small cell lung carcinomas: frequency and prognostic significance. *Eur J Cancer* **30A**: 376–381

Reinhardt KM, Steiner M, Zillig D, Nagel HR, Blann AD and Brinckmann W (1996) Soluble intercellular adhesion molecule 1 in colorectal cancer and its relationship to acute phase proteins. *Neoplasma* **43**: 65–67

Roit I, Brostoff J and Male D (1996) *Immunology*, 4th edn. Mosby: London

Rothlein R, Mainolfi EA, Czaikowski M and Marlin SD (1991) A form of circulating ICAM-1 in human serum. *J Immunol* **147**: 3788–3739

Schadendorf D, Diehl S, Zuberbier T, Schadendorf C and Henz BM (1996) Quantitative detection of soluble adhesion molecules in sera of melanoma patients correlates with clinical stage. *Dermatology* **192**: 89–93

Shimizu Y, Minemura M, Tsukishiro T, Kashii Y, Miyamoto M, Nishimori H, Higuchi K and Watanabe A (1995) Serum concentration of intercellular adhesion molecule 1 in patients with hepatocellular carcinoma is a marker of the disease progression and prognosis. *Hepatology* **22**: 525–531

Sung KL, Kuhlmann P, Maldonado F, Lollo BA, Chien S and Brian AA (1992) Force contribution of the LFA-1/ICAM-1 complex to T cell adhesion. *J Cell Sci* **103**: 259–266

Van den Stolpe A and van der Saag PT (1996) Intercellular Adhesion Molecule 1. *J Mol Med* **74**: 13–33

Vora AR, Rodgers S, Parker AJ, Start R, Rees RC and Murray AK (1997) An immunohistochemical study of altered immunomodulary molecule expression in head and neck squamous cell carcinoma. *Br J Cancer* **76**: 836–844

Welder CA, Lee DH and Takei F (1993) Inhibition of cell adhesion by microspheres coated with recombinant soluble intercellular adhesion molecule 1. *J Immunol* **150**: 2203–2210

Wolff JM, Stephenson RN, Chisholm GD and Habib FK (1995) Levels of circulating intercellular adhesion molecule 1 in patients with metastatic cancer of the prostate and benign prostate hyperplasia. *Eur J Cancer* **31A**: 339–341

© Cancer Research Campaign 1999
